# Methylation dependent down-regulation of *G0S2* leads to suppression of invasion and improved prognosis of *IDH1*-mutant glioma

**DOI:** 10.1371/journal.pone.0206552

**Published:** 2018-11-02

**Authors:** Takanori Fukunaga, Yuki Fujita, Haruhiko Kishima, Toshihide Yamashita

**Affiliations:** 1 Department of Neurosurgery, Osaka University Graduate School of Medicine, 2–2 Yamadaoka, Suita, Osaka, Japan; 2 Department of Molecular Neuroscience, Osaka University Graduate School of Medicine, 2–2 Yamadaoka, Suita, Osaka, Japan; 3 Immunology Frontier Research Center, Osaka University, 3–1 Yamadaoka, Suita, Osaka, Japan; 4 Graduate School of Frontier Biosciences, Osaka University, 1–3 Yamadaoka, Suita, Osaka, Japan; University of Navarra, SPAIN

## Abstract

Isocitrate dehydrogenase (*IDH*) mutations are a prognostic factor in diffuse glioma. However, the mechanism by which these mutations improve prognosis are not clear. In a subset of *IDH*-mutant glioma, remodeling of the methylome results in the glioma-CpG island methylator phenotype (G-CIMP) and transcriptional reorganization. In this study, we focus on G0/G1 switch 2 (*G0S2*), which is highly downregulated in G-CIMP glioma. We found that *G0S2* expression tended to increase as the WHO grade increased, and *G0S2* knockdown inhibited glioma invasion. Additionally, we revealed that the overexpression of the DNA demethylase Ten-eleven translocation 2 (*TET2*) in *IDH1*-plasmid transfected glioblastoma multiforme (GBM) cells restored *G0S2* expression. These results indicate that *G0S2* is epigenetically silenced in *IDH1*-mutant glioma. In addition, the stereotactic delivery of glioma cells with decreased *G0S2* expression in the mouse brain resulted in prolonged survival. The Cancer Genome Atlas (TCGA) analysis also indicated that survival is longer in the lower *G0S2* expression group than in the higher *G0S2* expression group. Moreover, *G0S2* expression was higher in recurrent tumor specimens than at the initial diagnosis in the same patient. These results provide one explanation for the improved survival in *IDH1*-mutant glioma as well as a new epigenetic target for glioma treatment.

## Introduction

Glioma, the most common primary tumor in the central nervous system, is classified into four grades according to its malignancy based on histopathological and molecular features established by the World Health Organization (WHO). WHO grade IV glioblastoma multiforme (GBM) is the most malignant and has the worst prognosis among all glioma. Although the current standard treatment includes the maximal removal of the tumor, followed by adjuvant chemotherapy and radiation therapy, the median overall survival is only 16.8 months [1]. Treatment approaches and survival have not improved for decades.

Moreover, as glioma cells invade eloquent areas and migrate undetectably toward normal outer brain tissues, it is difficult to resect the entire glioma by surgery. Accordingly, most gliomas recur and progress to more malignant WHO grades than that at primary diagnosis. Unfortunately, there is no standard therapy for recurrent glioma. Thus, new treatment targets are urgently needed.

Most GBM (~90%) occur in elderly people and develop rapidly (primary GBM or *de novo* GBM). However, a subset of GBM that progresses from a lower grade (WHO grade II/III) glioma (termed secondary GBM) occurs in younger people and has a significantly better prognosis than primary GBM [2, 3, 4].

Although primary and secondary GBM are indistinguishable in terms of pathological findings, they have different genomic backgrounds. Genome-wide analyses have shown that novel somatic mutations in isocitrate dehydrogenase 1 (*IDH1*) occur in 12% of all patients with GBM [5]. An *IDH1* mutation at the arginine residue in codon 132, most commonly the R132H mutation [5, 6], promotes the direct catalysis of α-ketoglutarate (α-KG) to 2-hydroxyglutarate (2-HG). 2-HG is a competitive inhibitor of α-KG-dependent dioxygenases, including histone demethylases and the Ten-eleven translocation (*TET*) family of 5-methlycytosine (5mC) hydroxylases and hence results in genome-wide DNA methylation [7, 8]. *IDH* mutations are enriched in WHO grade II and III astrocytoma and oligodendroglioma and in secondary GBM (>75%), but are not common in primary GBM (5%) [6, 9, 10, 11].

In addition, the updated fourth edition of the WHO Classification of CNS Tumors published in 2016 includes well-established molecular genetic parameters for subclassification [12]. In this classification, *IDH1* mutations, the codeletion status of chromosome arms 1p and 19q, and the histone 3 mutational status can be used to distinguish between biologically distinct glioma [13, 14]. Moreover, it has been reported that the genomic status better predicts outcome than histologic grade [15].

Patients with WHO grade III anaplastic astrocytoma and GBM carrying *IDH1* mutations have a significantly longer median overall survival than patients with wild-type *IDH1* [6]. However, the mechanism underlying the improved prognosis in patients with glioma carrying *IDH1* mutations is not fully understood.

Concurrent genome-wide DNA methylation and DNA promoter CpG island hypermethylation leads to transcriptional silencing in cancer [16]. The frequency of promoter methylation is higher in secondary GBM than in primary GBM [17]. A distinct subset of glioma displaying DNA hypermethylation, i.e., glioma-CpG island methylator phenotype (G-CIMP), has a characteristic profile; it is more prevalent among lower grade glioma, strongly associated with *IDH1* mutation, diagnosed at a younger age, and has a significantly better prognosis [18]. However, the functional importance of this altered epigenetic state remains unclear. In comparisons between G-CIMP-positive tumors and G-CIMP-negative tumors, G0/G1 switch 2 (*G0S2*) exhibits the strongest differential expression [18], yet the mechanism and function of *G0S2* in glioma are unclear. These observations prompted us to hypothesize that the epigenetic silencing of *G0S2* in G-CIMP may improve prognosis in patients with *IDH1* mutations. In this study, we revealed the functional role of *G0S2* in glioma and elucidated one explanation for the increased survival in glioma with *IDH1* mutation.

## Materials and methods

### Cell culture

U87 and U251 cells were purchased from the American Type Culture Collection (ATCC, Manassas, VA, USA) and cultured at 37°C in Dulbecco's Modified Eagle Medium (DMEM) supplemented with 10% fetal bovine serum. Normal human astrocytes were obtained from Gibco (N7805-100, Gaithersburg, MD, USA) and cultured in complete astrocyte medium according to the manufacturer’s instructions.

### RNA preparation and qRT-PCR analysis

Total RNA was prepared using the RNeasy Mini Kit (74104; Qiagen, Hilden, Germany) or TRIzol Reagent (15596026; Invitrogen, Carlsbad, CA, USA) according to the manufacturers’ instructions. Total RNA was converted into cDNA using the High Capacity cDNA Reverse Transcription Kit (4368814; Thermo Fisher, Waltham, MA, USA) and amplified according to the manufacturer’s protocol. RT-PCR was performed using Fast SYBR Green Master Mix (4385612; Thermo Fisher), with β-actin as an internal control, using the following primers: *G0S2* (#1; forward 5′-CCGCTGACATCTAGAACTGACCTA-3′ and reverse 5′-CAGCAAAACTCAATCCCAAACTC-3′, #2; forward 5′-GCTGACATCTAGAACTGACCTA-3′ and reverse 5′- CAGCAAAACTCAATCCCAAACTC-3′) and β-actin (forward 5′- GCGGGAAATCGTGCGTGACA-3′ and reverse 5′-AAGGAAGGCTGGAAGAGTGC-3′).

### Analysis of *G0S2* expression in tumor samples

Either signed consent forms or exemptions were obtained for all human samples. The study of human samples was approved by the Osaka University Institutional Review Board (17022–3). Tumor specimens were obtained during surgery and classified according to the histological grade of nervous system tumors published by the WHO in 2007. All tissue samples used in this study were acquired with informed consent, and all experiments were performed in accordance with relevant guidelines and regulations of the Graduate School of Medicine of Osaka University. Human astrocytes were used as normal controls. Gene expression levels were analyzed by qRT-PCR, as described above.

### siRNA/plasmid transfection

siRNA/plasmid was transfected into U251 using Lipofectamine 2000 (Invitrogen) according to the manufacturer's instructions. Gene expression levels were analyzed by qRT-PCR, as described above. *G0S2* siRNAs used were (Invitrogen #147175, #147176, and #181705, referred to as #1, #2, and #3 in this study). G0S2 siRNA #1 used, unless otherwise specified.

On day 0, U251 cells were plated in a 60-mm dish and cultured at 37°C. On day 1, cells were 70–90% confluent, and siRNA/plasmid transfection was performed. On day 3, cells were extracted.

### Construct of *G0S2* plasmid

The *G0S2* sequence was amplified from U251 cDNA as a template using the following primers: (forward 5′- GGGAAGATGGTGAAGCTGTA -3′ and reverse 5′- CTGGTCTCCCACAGTTCCTA -3′). After ligation with pGEM-T Easy Vector (A1360; Promega, Wisconsin Madison, USA), G0S2 cds was amplified at the BglII site and EcoRI site with the following primers: (forward 5′- CGCAGATCTATGGAAACGGTCCAGGAGCTG -3′ and reverse 5′- AATGAATTCGGGAGGCGTGCTGCCGGTTGG -3′). The resulting double-stranded oligo-DNA was subcloned to the pAcGFP1-N1 vector (632469; Takara Bio Inc, Japan) at the BglII and EcoRI sites. The insert was confirmed by both restriction enzyme digestion and DNA sequencing.

### Rescue experiment for *G0S2* siRNA

Using the above *G0S2* overexpression plasmid as a template, a siRNA-resistant form of *G0S2* was generated by changing the targeted sequence of the siRNA to 5′-AAATGGTCAAACTGTATGT-3′ (4 mutant nucleotides are underlined) using the KOD-Plus-Mutagenesis Kit (Toyobo, Osaka, Japan). The plasmid was confirmed by DNA sequencing.

### Production of a lentiviral vector expressing *G0S2* shRNA

The shRNA sequence was made using *G0S2* siRNA sense and antisense sequences with one nucleotide exchanged at the BglII site (GATCT -> GATCC) and XbaI site (*G0S2* shRNA: 5′-GATCCCCAGATGGTGAAGCTGTACGTACGTGTGCTGTCCGTACGTACAGCTTCACCATCTTTTTTGGAAAT, control shRNA: 5′-GATCCCCACTACCGTTGTTAAGGTGACGTGTGCTGTCCGTCACCTTAACAACGGTAGTTTTTTGGAAAT) and their complementary strands were synthesized (Fasmac) and annealed in TE buffer by heating to 100°C for 2–3 min followed by cooling to room temperature. The resulting double-stranded oligo-DNA was subcloned to the pENTR4-H1 entry vector at the BglII and XbaI sites. The insert was confirmed by both restriction enzyme digestion and DNA sequencing. Next, pENTR4-H1-shRNA was cloned into the lentiviral vector (CS-RfA-CG) using Gateway LR Clonase II Enzyme Mix (Invitrogen) according to the manufacturer’s instructions. The insert was confirmed by both restriction enzyme digestion and DNA sequencing. These control or *G0S2* shRNA-expressing lentiviral vector stocks were generated by the transfection of 293T cells. The 293T cells were cultured in DMEM supplemented with 10% fetal bovine serum. 293T cells were plated in 100-cm^2^ dishes in 10 ml of the medium and transfected the following day with 5 μg of the pCAG-HIV plasmid, 5 μg of the pCMV-VSV-G-RSV-Rev plasmid, and 10 μg of each lentiviral vector plasmid. Lentiviral vectors were collected on day 5 post-transfection and filtered through a 0.45-μm pore size filter and concentrated by ultracentrifugation at 4°C for 2 h at 48,820 × *g*.

U87 cells were plated on 100-cm^2^ dishes at a density of 1 × 10^6^ cells in 9 ml of medium per dish. Transductions were carried out in the presence of 4 μg/ml Polybrene for Lenti-control or *G0S2* shRNA. After incubation for 12 h, the transduction medium was replaced with fresh DMEM.

### Transwell invasion assay

Transwell inserts (6.5 mm, 8-μm pore size membrane) were coated with vitronectin (10 μg/mL in PBS). The assay was largely performed as previously reported [19]. At 2 days after siRNA transfection, U251 cells were harvested with Trypsin-EDTA (0.25%), washed with PBS, and resuspended in invasion assay buffer at 4 × 10^5^ cells/ml. Cell aliquots of 100 μl were plated on the upper filter surface and incubated at 37°C for 3 h. Filters were then washed, and cells on the upper surface were removed using cotton swabs. Cells on the lower filter surface were fixed in 4% paraformaldehyde for 30 min, blocked in PBS with 0.1% Triton-X and 5% BSA for 1 h, and stained with DAPI. Five random selected fields were evaluated with a 100× oil immersion lens; the number of invaded cells was compared with cells counts in the control group. An average of five fields at 100× total magnification was used for the quantitative analysis.

### In vivo tumorigenicity assay

All experiments were performed with the approval of the Animal Studies Ethics Committee of the Osaka University Hospital (J4822), and all experiments were performed in accordance with the Guide for the Care and Use of Laboratory Animals of the Graduate School of Medicine of Osaka University. Five-week-old NOD-SCID mice (Charles River) were randomly divided into two groups (three mice per group). Under anesthesia, a 1-mm burr hole was made (2 mm to the midline and 2 mm posterior to the bregma) using a microskull drill. A total of 2 × 10^5^ cells (U87 transfected with Lenti-control or *G0S2* shRNA) suspended in 1 μl of PBS were slowly and smoothly injected to a depth of 3 mm via the skull hole using a 10-μl Hamilton syringe with a 30-gauge needle at a rate of 0.5 μl/min into the subcortex of the mouse brain. The needle was retained in place for 2 min. The scalp was sutured. Survival was recorded every 2 days over the course of the study.

At 4 weeks post-injection, anesthetized mice were perfused. The brains were removed and fixed in 4% paraformaldehyde for 24 h and then cryoprotected in 30% sucrose. Frozen coronal sections (30 μm) were made. For immunofluorescence, sections were incubated overnight at 4°C with the rabbit monoclonal anti-GFP antibody (1:1000 dilution, A-11122; Invitrogen). Following washing, the sections were treated with Alexa Fluor 488 goat anti-rabbit IgG (H+L) secondary antibody (1:1000 dilution, A-11008; Invitrogen).

### Immunohistochemistry for *G0S2*

Immunohistochemical analyses of *G0S2* were performed using paraffin blocks of specimens. The sections were deparaffinized in xylene and rehydrated in a descending ethanol-to-water gradient series. Endogenous peroxidase was blocked by exposure to 1% H_2_O_2_ at room temperature for 1 h. Sections were soaked for antigen retrieval in pH 6.0 citrate buffer at 125°C for 5 min. After cooling to room temperature, sections were blocked with 10% goat serum in PBS for 30 min at room temperature. Sections were incubated for 2 h at room temperature with a primary antibody against *G0S2* (1:200; Novus, St. Louis, MO, USA) followed by incubation for 30 min at room temperature with Anti-rabbit IgG labeled with biotin (1:1000). The sections were prepared for the chromogen reaction with 0.05% diaminobenzidine and 0.01% H_2_O_2_, and were counterstained with hematoxylin.

### Analysis of the cancer genome Atlas dataset

For the WHO grade II–IV glioma analysis, *G0S2* gene expression based on RNASeq data (TOIL RSEM log2(normalized_count+1)), DNA methylation β values (the ratio of methylation-specific and demethylation-specific fluorophores), average Methylation450K data, *IDH1* binary non-silent mutation (broad), primary tumor (sample_type) for brain lower grade glioma (LrGG, WHO grade II/III), and glioblastoma multiforme (GBM, WHO grade IV) samples were extracted from the TCGA Pan-Cancer (PANCAN) dataset using the University of California Santa Cruz Xena Browser (http://xena.ucsc.edu/). Excluding null data, 554 matching samples were available (509 lower grade glioma and 45 primary GBM, 157 wt *IDH1* and 397 mut *IDH1*). For the lower grade (WHO grade II/III) glioma analysis, *G0S2* gene expression based on RNASeq (polyA+ IlluminaHiSeq) data, DNA methylation β values, average Methylation450K data, *IDH1* mutation found (0 = No, 1 = Yes), WHO grade II/III (neoplasm histologic grade), and primary tumor (sample_type) samples were extracted. Excluding null data, 125 matching samples were obtained (57 WHO grade II and 68 WHO grade III), dividing into two groups according to the G0S2 expression level (“G0S2 low” and “G0S2 high”). The survival ratio was compared with respect to *G0S2* expression within the same WHO grade. For the GBM analysis, *G0S2* gene expression based on RNASeq (polyA+ IlluminaHiSeq) data and primary tumor (sample_type) samples were extracted from the TCGA Glioblastoma (GBM) dataset. Excluding null data, 154 matching samples were obtained, the survival ratio was compared with respect to *G0S2* expression among the GBM samples.

Additionally, the G0S2 mutation status was extracted from the glioma dataset {Merged Cohort of LGG and GBM (TCGA, Cell 2016)} using cBioPortal for Cancer Genomics (www.cbioportal.org), and 1102 samples were available.

### Statistical analysis

Data are presented as means ± SEM (standard error of the mean). Comparisons between groups were performed using Student’s *t*-tests and one-way ANOVA followed by Tukey–Kramer tests. Statistical significance was set at *p* < 0.05. JMP statistical package was used for the statistical analyses.

The statistical analyses of TCGA datasets using Welch's t-test, Pearson's and Spearman's rank correlation were collected from the University of California Santa Cruz Xena Browser (http://xena.ucsc.edu/)

## Results

### Glioma with low *G0S2* expression is associated with a longer overall survival in the TCGA dataset

**Fig 1:**
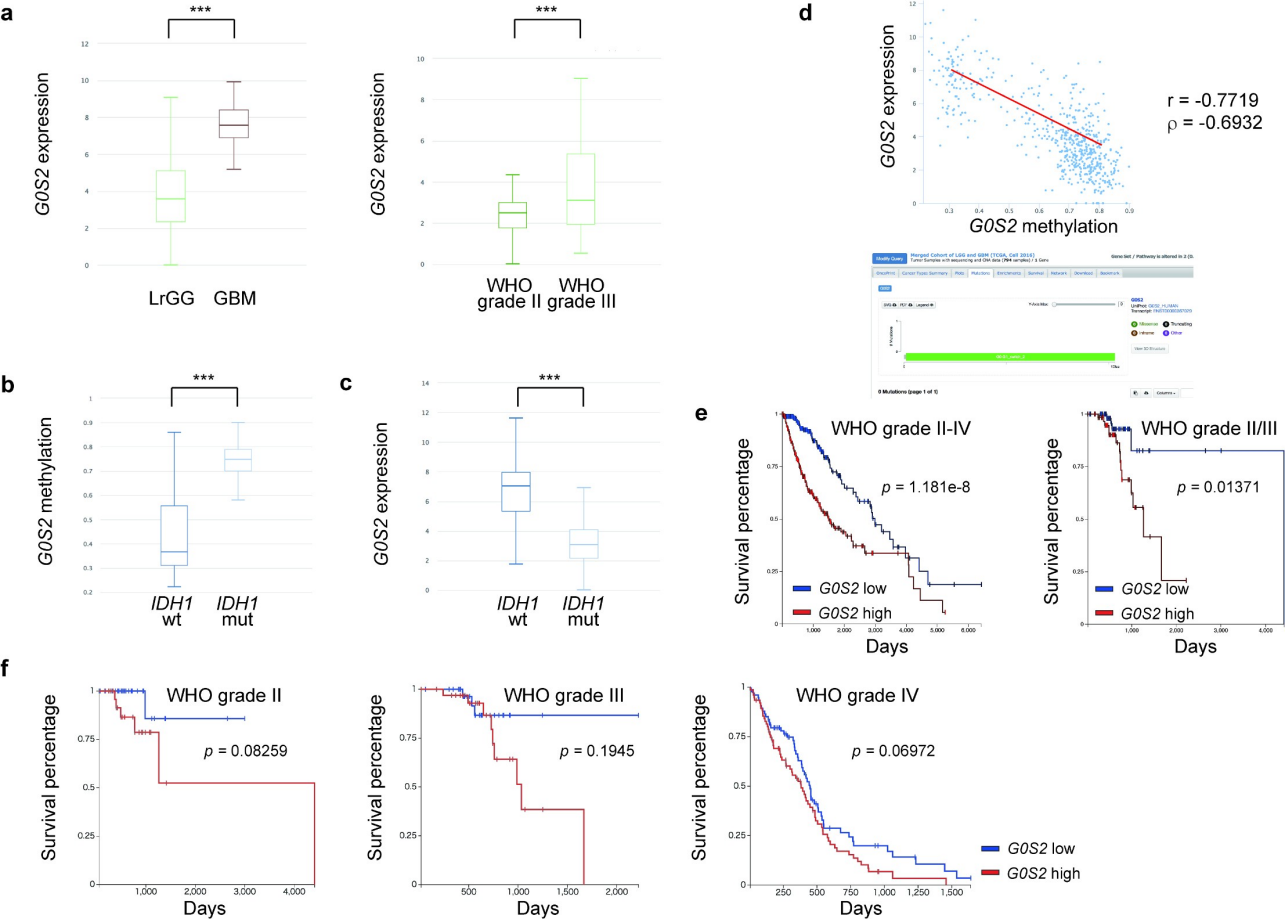
Analysis of the TCGA dataset. (**a**) As the WHO grade increased, *G0S2* expression significantly increased. (left) *G0S2* expression was higher in primary GBM (7.57, n = 45) than in lower grade gliomas (LrGG, WHO grade II/III, 3.59, n = 509). (right) *G0S2* expression was higher in WHO grade III (3.12, n = 68) than WHO grade II (2.49, n = 57). ***p < 0.001 (*p* = 1269e-16, left; *p* = 0.00633, right) using Welch’s *t*-test. (**b**) The *G0S2* methylation β values were higher with *IDH1* mutations (mut) (0.749, n = 397) than with wild-type (wt) *IDH1* (wt) (0.366, n = 157) among WHO grade II–IV gliomas. ***p < 0.001 (*p* = 9.439e-53) using Welch’s *t*-test. (**c**) G0S2 expression was lower in samples with *IDH1* mutations (3.1, n = 397) than wild-type *IDH1* (7.03, n = 157) among WHO grade II–IV gliomas. ***p < 0.001 (*p* = 0.000). Welch’s *t*-test. (**d**) (upper) *G0S2* gene expression was negatively associated with *G0S2* methylation (Pearson’s *r* = -0.7719, Spearman’s rho = -0.6932). (lower) There were no *G0S2* mutations in 1102 gliomas among the TCGA WHO grade II–IV glioma cohort. (**e**) (left) The lower *G0S2* expression group (n = 273) clearly showed better overall survival than the higher G0S2 expression group (n = 275) in WHO grade II–IV glioma. Log-rank test, *p* = 1.181e-8. (right) The lower *G0S2* expression group (n = 63) clearly showed better overall survival than the higher *G0S2* expression group (n = 62) in WHO grade II/III glioma. Log-rank test, *p* = 0.01371. (**f**) The lower *G0S2* expression group tended to have a better prognosis than the higher *G0S2* expression group within the same WHO grade glioma (left, WHO grade II, n = 28, 29; middle, WHO grade III, n = 34, 34; right, WHO grade IV, n = 76, 76, where n = lower expression, higher expression).

Using the Cancer Genome Atlas (TCGA) database, we found that *G0S2* expression elevated as the WHO grade increased among WHO grade II–IV gliomas. Relative *G0S2* expression was higher in GBM than in lower grade gliomas (7.57 vs. 3.59, *p* = 1269e-16), and was higher in WHO grade III glioma than in WHO grade II glioma (3.12 vs. 2.49, *p* = 0.006330) among the lower grade gliomas (Fig 1A). Mutant *IDH1* was statistically significantly related to both higher *G0S2* methylation (0.749 vs. 0.366, *p* = 9.439e-53) and lower *G0S2* expression (3.1 vs. 7.03, *p* = 0.000) compared to wild-type *IDH1* among WHO grade II–IV gliomas (Fig 1B and 1C). *G0S2* gene expression was negatively associated with *G0S2* methylation (Pearson’s *r* = -0.7719, Spearman’s rho = -0.6932) within WHO grade II–IV gliomas (Fig 1D). Interestingly, *G0S2* mutations were not reported in 1102 gliomas among TCGA WHO grade II–IV gliomas (Fig 1D), supporting the notion that *G0S2* is epigenetically silenced in gliomas harboring *IDH1* mutations. In addition, the lower *G0S2* expression group clearly showed better over survival than the higher *G0S2* expression group for all WHO grade II–IV gliomas (*p* = 1.181e-8) and for WHO grade II/III lower grade gliomas (*p* = 0.01371) (Fig 1E). Furthermore, the lower *G0S2* expression group tended to exhibit a better prognosis than the higher *G0S2* expression group among the same WHO grade (Fig 1F). These observations suggest that *G0S2* is a new potential diagnostic marker for glioma.

### Decreased *G0S2* expression in glioma with *IDH1* mutation

**Fig 2:**
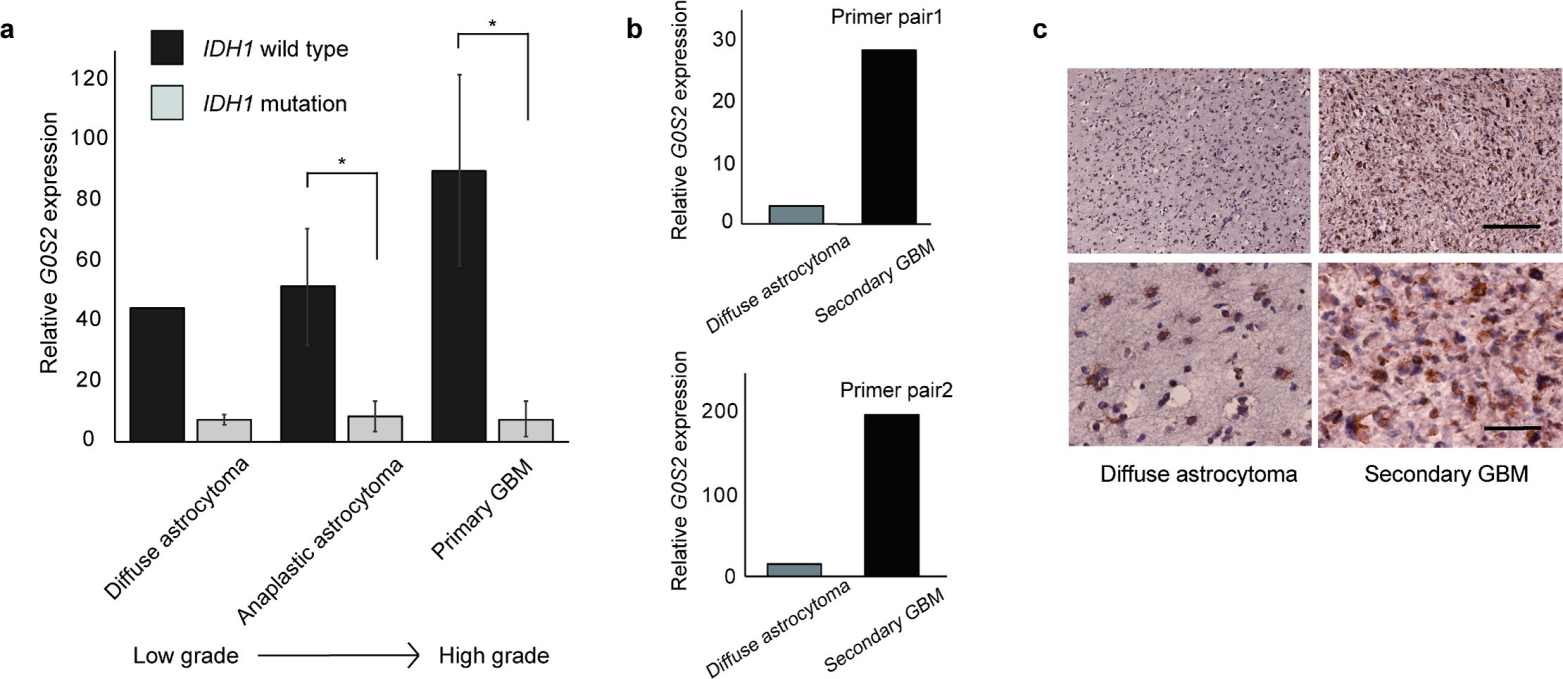
Relative *G0S2* expression was downregulated in gliomas with mutant *IDH1*. (**a**) Relative G0S2 expression by RT-PCR was quantitatively analyzed in primary glioma samples (n = 26). The data were normalized by β-actin, used as the internal control, followed by normalization by cultured human astrocyte. *G0S2* mRNA was significantly higher in gliomas with wild-type *IDH1* than with mutant *IDH1* for WHO grade III/IV glioma. Although there was no significant difference owing to the limited specimen number, *G0S2* mRNA tended to increase as the WHO grade increased. Wild-type (wt) *IDH1* diffuse astrocytoma, n = 2; mutant (mut) *IDH1* diffuse astrocytoma, n = 3; wt *IDH1* anaplastic astrocytoma, n = 3; mut *IDH1* anaplastic astrocytoma, n = 5; wt *IDH1* primary GBM, n = 10; mut *IDH1* primary GBM, n = 3. *p < 0.05, Student’s *t*-test. **(b)** *G0S2* expression was higher at the recurrent diagnosis than at the initial diagnosis in the same patient. Relative *G0S2* expression was quantified by RT-PCR using normal human astrocytes as a control. *G0S2* expression was elevated in recurrent glioma, diagnosed as WHO grade IV secondary GBM, compared to initial glioma, diagnosed as WHO grade II diffuse astrocytoma, in the same patient. β-Actin was used as an internal control. n = 1. (**c**) Representative images of the sections of diffuse astrocytoma (DA) and secondary GBM (sGBM) immunostained with an anti-*G0S2* antibody. The specimens were prepared from the same case. *G0S2* expression was higher in sGBM than in DA. Scale bar: 200 μm (low-magnification, upper panel), 50 μm (high-magnification, lower panel).

To verify the role of *G0S2* in glioma malignancy, we measured *G0S2* gene expression in WHO grade II diffuse astrocytoma, grade III anaplastic astrocytoma, and grade IV primary GBM tissue samples (Fig 2A). *G0S2* levels were significantly higher for wild-type *IDH1* than for mutant *IDH1* among WHO grade III/IV gliomas. Although the correlation was not analyzed owing to the limited number of samples (n = 2, wild-type *IDH1* WHO grade II specimens), *G0S2* mRNA levels tended to be elevated as the WHO grade increased. These findings suggest that reduced expression of *G0S2* contributes to better pathological features in glioma with *IDH1* mutation.

Further, mRNA expression of *G0S2* was higher in recurrent malignant glioma, diagnosed as WHO grade IV secondary GBM, than in initial glioma, diagnosed as WHO grade II diffuse astrocytoma, within the same patient (Fig 2B). *G0S2* mRNA expression was higher in the same human high-grade glioma tissues (secondary GBM) than in low-grade glioma tissues (diffuse astrocytoma; DA) (Fig 2C). These results suggest that increased *G0S2* gene expression is associated with the malignancy of glioma.

### Knockdown of *G0S2* reduces the invasion of glioma cells

**Fig 3:**
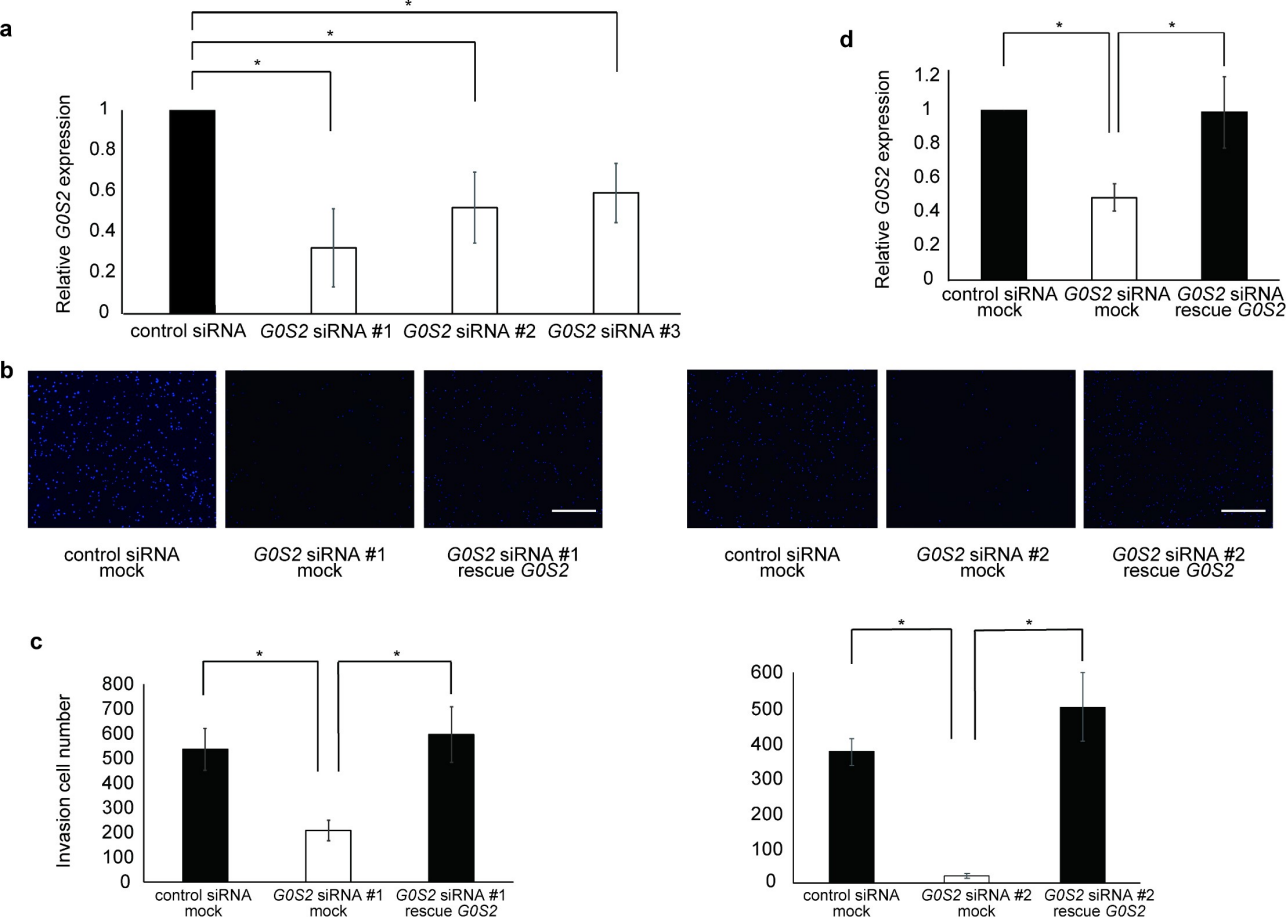
Repression of *G0S2* expression inhibits glioma cellular invasion. (**a**) Each *G0S2* siRNA knocked down gene expression. n = 3. *p < 0.05 using Student’s *t*-test. (**b**) The cells were transfected with the indicated siRNA #1, #2 and plasmid and invasion ability was measured using transwell assays. Representative images of the cells stained with DAPI are shown. (**c**) The number of invaded cells was determined. (**d**) Invasion ability was blocked by *G0S2* siRNA, and re-exacerbated by *G0S2* rescue. n = 5. *p < 0.05 by one-way ANOVA followed by a Tukey–Kramer test. Scale bar: 200 μm.

Next, we analyzed the role of *G0S2* in glioma invasion by a transwell assay. To perform loss-of-function experiments using small interfering RNA (siRNA), we examined the knockdown efficacy of *G0S2* siRNA. Efficient *G0S2* mRNA downregulation was observed in *G0S2* siRNA #1-, 2-, and 3-transfected cells, but not in the control, non-target siRNA-transfected cells (Fig 3A). siRNA-mediated knockdown of *G0S2* in the U251 malignant glioma cell line significantly suppressed cellular invasion (Fig 3B and 3C). To determine the specificity, the cells were transfected with both *G0S2* siRNA and the RNA interference (RNAi)-resistant *G0S2* rescue plasmid, and *G0S2* expression was recovered to the control level (Fig 3D). Reconstituting *G0S2* expression with the *G0S2* rescue plasmid restored the cellular invasion ability (Fig 3B and 3C). These results indicate that the inhibition of *G0S2* expression suppresses the invasion ability of glioma cells.

### Antitumor effect of *G0S2* knockdown in U87 glioma xenograft-bearing mice

**Fig 4:**
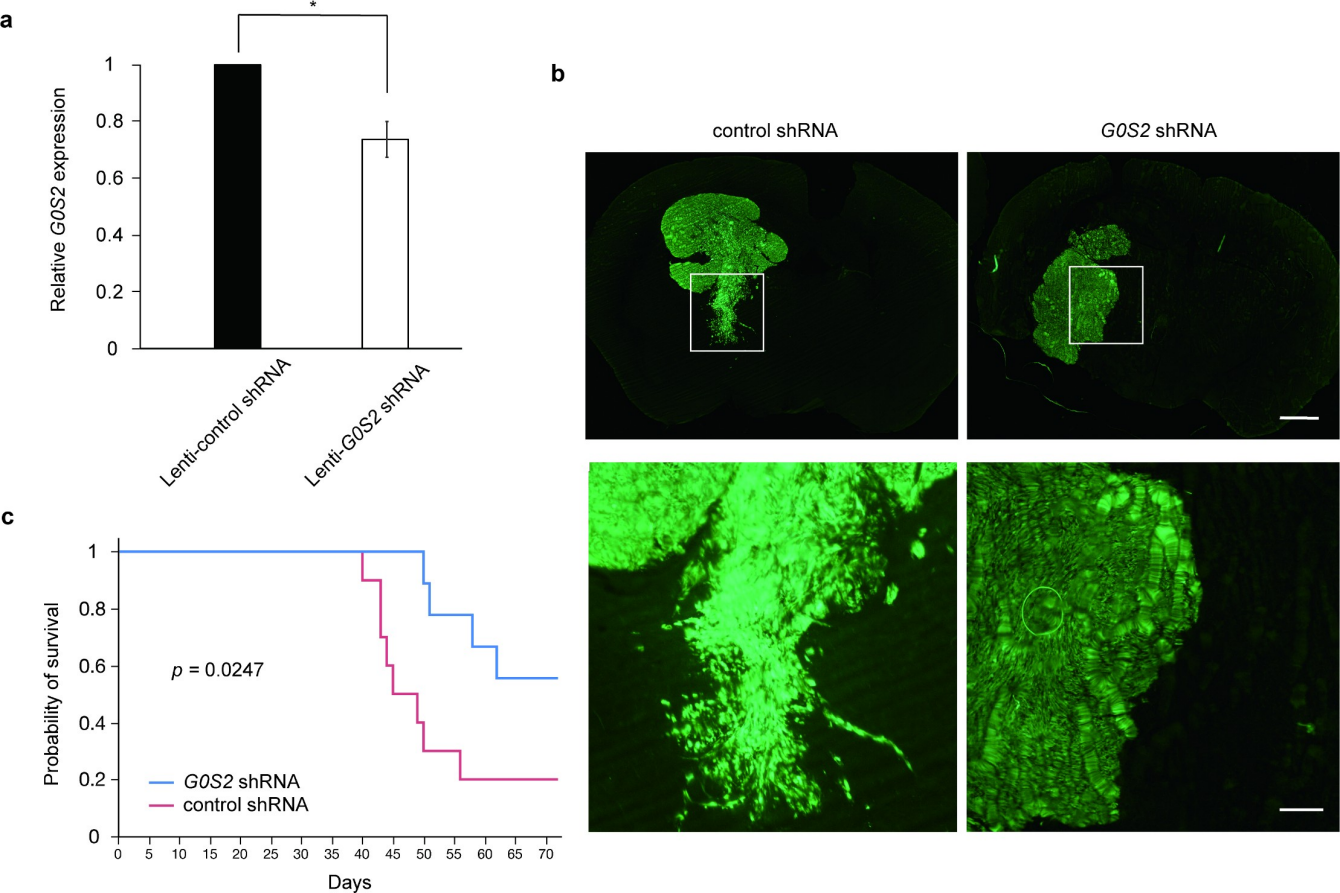
Suppression of *G0S2* improved the survival rate in a glioma xenograft model. (**a**) Lenti-*G0S2* shRNA-infected U87 with knocked down gene expression. n = 3. *p < 0.05 by Student’s *t*-test. (**b**) Although Lenti-*G0S2* shRNA-GFP U87 cells infiltrated normal brain tissues, the outer rim of Lenti–control shRNA–GFP U87 cells became smooth. Regions of interest are enlarged in the lower panel. Scale bar: 1 mm (low-magnification, upper panel), 200 μm (high-magnification, lower panel). (**c**) Mice injected with U87 cells with lentiviral expression of *G0S2* shRNA-GFP exhibited prolonged overall survival compared with those with control shRNA-GFP. Median OS; *G0S2* shRNA (n = 9) 64.6 days, control shRNA (n = 10) 51.4 days. *P* = 0.0247 by log rank tests.

Our *in vitro* experiments indicated that the downregulation of *G0S2* expression can efficiently attenuate the invasion of glioma cells. Therefore, we further examined the antitumor effect of *G0S2 in vivo* using a xenograft model. We first transplanted both U251 and U87 cells into mice brains, and found that U87 cells, but not U251 cells, tended to survive in the brain. We examined the knockdown efficacy of Lenti-*G0S2* shRNA in U87 cells (Fig 4A). U87 GBM cells infected with the lentivirus encoding *G0S2* shRNA in mouse brains exhibited reduced cell invasion toward surrounding normal brain tissues (Fig 4B). Importantly, mice in which *G0S2-*silenced GBM cells were injected into the brain exhibited prolonged overall survival (Fig 4C).

### *IDH1* mutation epigenetically represses the transcription of *G0S2*

**Fig 5:**
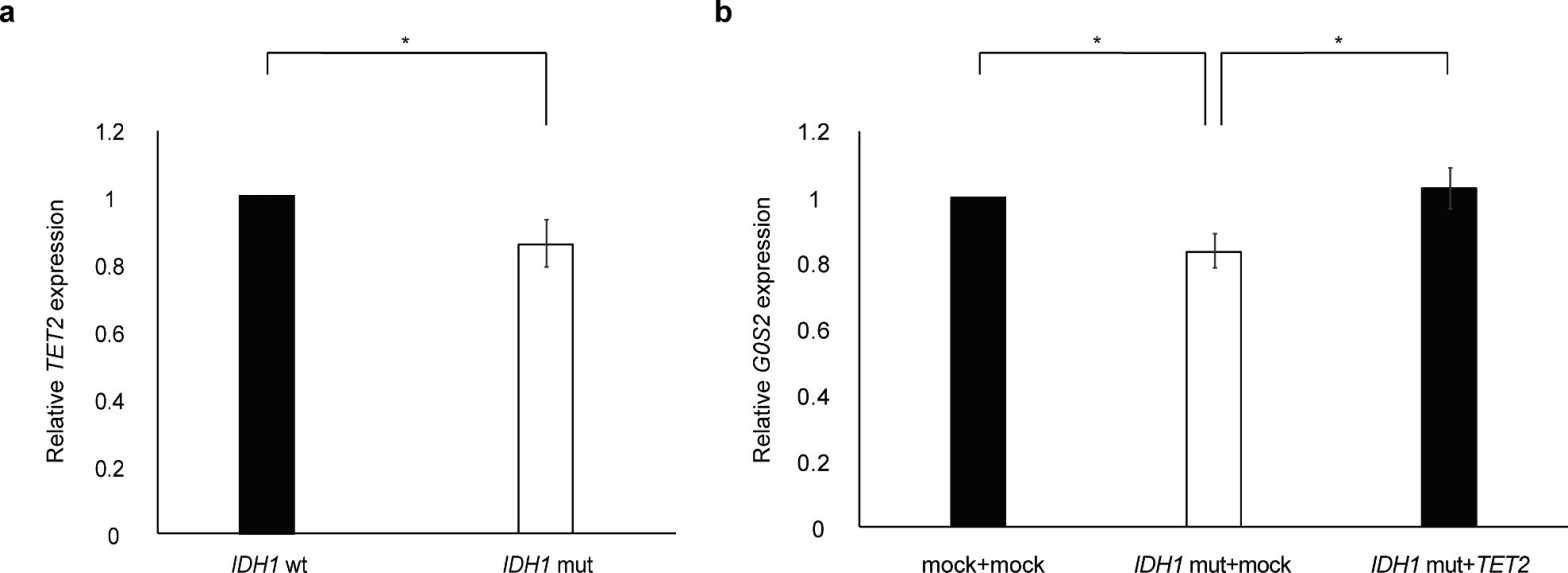
*G0S2* is epigenetically silenced in *IDH1* mutant glioma cells. (**a**) TET2 expression was decreased in U251MG glioma cells transfected with the IDH1 R132H mutant (mut) compared with wild-type (wt) IDH1. n = 5. *p < 0.05 by Student’s *t-*test. (**b**) After IDH R132H plasmid transfection, on day 2, cells were extracted. Quantification of relative *G0S2* expression by RT-PCR. Although the forced induction of the *IDH1* mutation plasmid to the U251 cells decreased *G0S2* expression, transfecting both the *IDH1* mutation plasmid and the *TET2* overexpression plasmid restored *G0S2* expression. The pcDNA-Flag plasmid was used as a mock plasmid.

Since 2-HG, which is increased in *IDH*-mutant glioma, directly inhibits the activity of *TET* DNA demethylase, many genes in cancer are epigenetically silenced by promoter hypermethylation [8,20]. Of note, the *G0S2* promoter contains a CpG island [21], suggesting that *G0S2* down-regulation in the mutant *IDH1* background results from epigenetic silencing. To test this hypothesis, we examined whether GBM cells transfected with a mutant *IDH1* plasmid (pcDNA3-Flag-IDH1-R132H, a gift from Yue Xiong, #62907; Addgene, Cambridge, MA, USA) decreased the *G0S2* mRNA level, and DNA hypomethylation by *TET2* overexpression could restore the *G0S2* mRNA level. It was reported that expression of *IDH1* mutation lead to decrease in 5hmC [22], and that 5hmC decrease was associated with *TET2* gene expression [23]. Actually, *TET2* expression was decreased in GBM cells transfected with the mutant *IDH1* plasmid (Fig 5A). Co-transfection of the mutant *IDH1* plasmid with the *TET2* plasmid (FH-TET2-pEF, a gift from Anjana Rao, Addgene #41710) recovered *G0S2* expression (Fig 5B). These results suggest that *IDH1* mutation represses *G0S2* by DNA methylation.

## Discussion

Mutations and the overexpression of several oncogenes have been identified in glioma [24, 25, 26]. The *IDH* R132H mutation, in particular, is thought to play a key role in gliomagenesis. This point mutation results not only in a loss of function, i.e., an inability to catalyze the conversion of isocitrate to α-ketoglutarate, but also in a gain of function, i.e., the ability to catalyze the NADPH-dependent reduction of α -ketoglutarate to 2-hydroxyglutarate (2-HG). Excess 2-HG contributes to the progression of epigenetic alterations, including DNA methylation at promoter CpG islands, and finally increases the risk of developing glioma [7]. Chao et al. reported that 2-HG accumulation can inhibit H3K9 demethylase *KDM4C* (also known as *JMJD2C*) and that the inhibition of histone demethylation impairs the differentiation of non-transformed cells into terminally differentiated cells [27]. In addition, Flavahan et al. found that *IDH* mutations promote gliomagenesis by disrupting the chromosomal topology and allowing aberrant regulatory interactions that induce *PDGFRA* expression [28]. Hypermethylation of the *O*^6^-methylguanine-DNA methyltransferase (*MGMT)* promoter-associated CpG island has been reported as a favorable prognostic indicator. *MGMT* encodes an *O*^6^-methylguanine methyltransferase that removes alkyl groups from the *O*-6 position of guanine. Patients with *MGMT* hypermethylation show sensitivity to alkylating agents, such as temozolomide, with improved outcomes [29]. Rivera et al. reported that the methylation status of the *MGMT* promoter also predicts the response to radiotherapy in the absence of adjuvant alkylating agents in patients with newly diagnosed GBM [30].

*IDH* mutations are associated with improved survival in patients with glioma [6], and are a useful prognostic marker in clinical settings. However, the mechanism underlying the improved prognosis in patients with *IDH* mutations has not been determined. In this study, we elucidated the role of *G0S2* in glioma cell invasion and identified a mechanism by which patients with glioma carrying *IDH* mutations and G-CIMP show better prognosis.

At present, only histopathological findings can be used to distinguish between WHO grade II and WHO grade III gliomas. According to our analysis, survival was longer for the lower *G0S2* expression group than for the higher *G0S2* expression group among the patients with WHO grade II–IV gliomas and WHO grade II/III gliomas in the TCGA dataset. Moreover, *G0S2* expression increased as the WHO grade increased. We also demonstrated that the expression of *G0S2* was higher in post-operative recurrence diagnosed as WHO grade IV secondary GBM compared with initial glioma diagnosed as WHO grade II diffuse astrocytoma in the same patient. It should be noted that there may be a contamination of normal brain parenchyma when using surgical material from low grade gliomas, resulting in lower relative G0S2 expression levels in the samples we examined. These data suggest that *G0S2* is correlated with malignant transformation and can be a new marker when glioma progresses to a higher WHO grade or recurrence. It is necessary to evaluate a larger cohort to validate the correlation between *G0S2* and WHO grade diagnosis. It is difficult to evaluate the mechanism underlying glioma malignant transformation, such as progression from WHO grade II/III to WHO grade IV, *in vitro* or *in vivo*; accordingly, an effective therapy for glioma recurrence is lacking. If a patient is diagnosed WHO grade II/III low-grade glioma, specifically suppressing *G0S2* methylation has the potential to prevent malignant transformation.

The inhibition of 2-HG has been proposed as a possible treatment strategy for *IDH*-mutant glioma [31]. Although DNA methyltransferase inhibitors and histone deacetylase inhibitors are in clinical trials and show promise for the treatment of hematopoietic malignancies [32, 33, 34], treatment with vorinostat, a histone deacetylase (HDAC) inhibitor, combined with standard chemoradiation in newly diagnosed GBM in a phase II cohort did not meet efficacy objectives [35, 36]. With respect to the molecular landscape, it is thought that focusing on specific alterations is more advantageous than conventional treatment strategies [37]. Our results further suggest that *G0S2* methylation may be a potential therapeutic target.

In other cancers, *G0S2* is correlated with cancer invasion [38], apotosis [39], and metabolism [40]. In a previous study of the genome-wide DNA methylation status of invasive cancer cell lines derived from breast, liver, and prostate cancers, *G0S2* mRNA was significantly upregulated compared to expression in non-invasive cell lines, and its depletion decreased cell invasion in these three invasive cell lines [38]. Previous study reported that U87 cells were used for analyzing invasion ability [41]. We demonstrated that glioma harboring mutant *IDH1* epigenetically represses *G0S2* expression, thereby suppressing surrounding cell invasion and resulting in a better prognosis. Consistent with our results, *G0S2* expression was down-regulated epigenetically by DNA hypermethylation in non-small cell lung cancer cell lines [38, 39]. *G0S2* is induced by tumor necrosis factor alpha (*TNFA*), whose activation also requires NFkB. *G0S2* promotes apoptosis by interacting with BCL2 and by preventing the formation of protective BCL2 and BAX heterodimers [39]. Kim et al. have demonstrated that the *G0S2* gene is significantly upregulated in *BRAF* V600E cells compared to wild-type *BRAF* human thyroid cells, and this expression change caused by the *BRAF* V600E mutation may have an important role in thyroid cancer development [42]. These observations are consistent with our results, demonstrating that *G0S2* downregulation is related to the suppression of glioma cell invasion (Fig 3).

Clearly, as the characteristics of glioma with *IDH* mutations are related to both the inactivation of several tumor suppressor genes and the overexpression of other genes, although it should be noted that *G0S2* is not an only marker for the better prognosis of *IDH* mutation glioma, the epigenetic down-regulation of the *G0S2* is not the only factor determining the biological characteristics of glioma, however, our results showed that epigenetic *G0S2* downregulation following *IDH1* mutation effectively inhibits the invasion of GBM cells both *in vitro* and *in vivo*. Unlike genetic mutations, epigenetic changes, such as promoter methylation, are reversible and can be targeted by drugs. Future studies should elucidate the downstream pathways involved in the suppression of invasion by *G0S2*.

In conclusion, we identified one mechanism by which *IDH* mutations improve prognosis in glioma. Continued investigations are necessary to shed light on the roles of *IDH* mutations.
